# Supplementary material for: Identification of photoperiod-induced specific miRNAs in the adrenal glands of Sunite sheep (Ovis aries)
Source: Front Vet Sci. 2022 Jul 22;9:888207. doi: 10.3389/fvets.2022.888207 (PMC9354845; doi:10.3389/fvets.2022.888207)
Supplement: Supplementary file 1 [file Data_Sheet_1.ZIP › Supplementary file/miRDeep2 file.zip!/miRDeep2 file/Novel_Structure/LP42A1/Novel_234.pdf]

Provisional ID	:	NC_019475.2_27804
Score total	:	1
Score for star read(s)	:	-1.3
Score for read counts	:	0
Score for mfe	:	0.7
Score for randfold	:	1.6
Score for cons. seed	:	
Total read count	:	104
Mature read count	:	104
Loop read count	:	0
Star read count	:	0

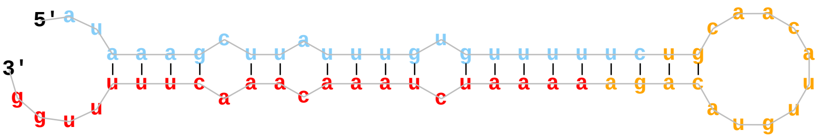

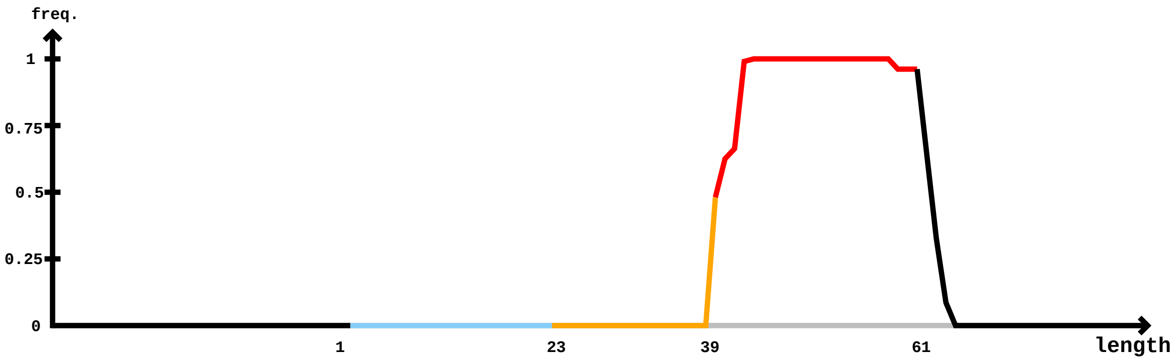

Star Mature

```
5'- uggccaugaagcuggccauaaagcuggccauaaagcuuauuuguguuuuucugcaacauuguacagaaaaaucuaaacaaacuuuuuggccaacccaauauuuuaucaaacu  -3'   exp
    ((((((......))))))......((((((.((((.((.((((.(((((((((.........))))))))).)))).)).)))).)))))).....................     reads      mm         sample
    ...................................................................aaaaucuGaacaaacuuuu..........................     4          1          seq
    ...................................................................aaaaucuGaacaaacuuuuugg.......................     45         1          seq
    ...................................................................aaaaucuGaacaaacuuuuuggc......................     1          1          seq
    ....................................................................aaaucuGaacaaacuuuuugg.......................     14         1          seq
    ....................................................................aaaucuaaacaaacuuuuuggc......................     1          0          seq
    .....................................................................aaucuGaacaaacuuuuuggc......................     4          1          seq
    ......................................................................aucuGaacaaacuuuuuggcc.....................     25         1          seq
    ......................................................................aucuaaacaaacuuuuuggcca....................     1          0          seq
    ......................................................................aucuGaacaaacuuuuuggcca....................     8          1          seq
    .......................................................................ucuGaacaaacuuuuuggc......................     1          1          seq
```
